# Analysis and cloning of the synthetic pathway of the phytohormone indole-3-acetic acid in the plant-beneficial *Bacillus amyloliquefaciens* SQR9

**DOI:** 10.1186/s12934-015-0323-4

**Published:** 2015-09-04

**Authors:** Jiahui Shao, Shuqing Li, Nan Zhang, Xiaoshuang Cui, Xuan Zhou, Guishan Zhang, Qirong Shen, Ruifu Zhang

**Affiliations:** Key Laboratory of Microbial Resources Collection and Preservation, Ministry of Agriculture, Institute of Agricultural Resources and Regional Planning, Chinese Academy of Agricultural Sciences, Beijing, 100081 People’s Republic of China; Jiangsu Key Lab and Engineering Center for Solid Organic Waste Utilization, Nanjing Agricultural University, Nanjing, 210095 People’s Republic of China

**Keywords:** *Bacillus*, Biosynthesis, Biotechnology, Phytohormone, Gene transcription, IAA biosynthesis pathway

## Abstract

**Background:**

The plant growth-promoting rhizobacteria (PGPR) strain *Bacillus amyloliquefaciens* SQR9, isolated from the cucumber rhizosphere, protects the host plant from pathogen invasion and promotes plant growth through efficient root colonization. The phytohormone indole-3-acetic acid (IAA) has been suggested to contribute to the plant-growth-promoting effect of *Bacillus* strains. The possible IAA synthetic pathways in *B*. *amyloliquefaciens* SQR9 were investigated in this study, using a combination of chemical and genetic analysis.

**Results:**

Gene candidates involved in tryptophan-dependent IAA synthesis were identified through tryptophan response transcriptional analysis, and inactivation of genes *ysnE*, *dhaS*, *yclC*, and *yhcX* in SQR9 led to 86, 77, 55, and 24 % reductions of the IAA production, respectively. The genes *patB* (encoding a conserved hypothetical protein predicted to be an aminotransferase), *yclC* (encoding a UbiD family decarboxylase), and *dhaS* (encoding indole 3-acetaldehyde dehydrogenase), which were proposed to constitute the indole-3-pyruvic acid (IPyA) pathway for IAA biosynthesis, were separately expressed in SQR9 or co-expressed as an entire IAA synthesis pathway cluster in SQR9 and *B. subtilis* 168, all these recombinants showed increased IAA production. These results suggested that gene products of *dhaS*, *patB*, *yclB*, *yclC*, *yhcX* and *ysnE* were involved in IAA biosynthesis. Genes *patB*, *yclC* and *dhaS* constitute a potential complete IPyA pathway of IAA biosynthesis in SQR9.

**Conclusions:**

In conclusion, biosynthesis of IAA in *B. amyloliquefaciens* SQR9 occurs through multiple pathways.

**Electronic supplementary material:**

The online version of this article (doi:10.1186/s12934-015-0323-4) contains supplementary material, which is available to authorized users.

## Background

Promotion of plant growth by *Bacillus* strains inhabiting the rhizosphere is well documented [1–4]. The plant growth-promoting effect of rhizospheric bacilli results from the synergy of several factors, including the production of phytohormones such as indole-3-acid (IAA), the secretion of extracellular phytase under conditions of phosphate limitation and in the presence of phytate [5, 6], and the emission of volatile compounds [2, 7, 8].

It appears that the secreted IAA plays an important role in the plant-growth-promoting effect of some plant-beneficial bacteria [9–11]. IAA is a common phytohormone with the capacity to regulate nearly every aspect of plant growth and development, such as cell division, elongation, fruit development and senescence [12–15]. IAA produced by plant-growth-promoting bacteria also acts in the rhizosphere, including stimulation of root-hair formation by increasing the number and length of lateral and primary roots when it is within a certain concentration range [16–18]. IAA synthesis in bacteria may lead to increased rooting, as observed in studies with *Azospirillum* mutants with altered IAA production [19]. This increased rooting enhanced plants’ water and nutrient uptake and root exudation, which in turn stimulated bacterial root colonization. A study by Ahmed and Hasnain [20] demonstrated that inoculation of two IAA-producing *Bacillus* strains could significantly increase the shoot length, root length and number of leaves on the plant compared to non-inoculated treatments, suggesting the possible use of IAA-producing bacteria as effective plant-growth-promoting inoculants.

The ability to synthesize IAA is a common trait in rhizospheric bacteria, and tryptophan has been identified as a main precursor for IAA biosynthesis pathways [21, 22]. To date, studies on the key genes or proteins involved in IAA production are few and have mostly been focused on a single specific gene or protein in a biosynthetic pathway. In plants, four tryptophan-dependent IAA synthesis pathways have been proposed, the indole-3-pyruvic acid (IPyA), indole-3-acetamide (IAM), indole-3-acetonitrile (IAN), and tryptamine (TAM) pathways, and they have also been postulated in bacteria [23, 24]. The IPyA pathway is thought to exist in both plants and bacteria, including phytopathogenic and beneficial bacteria [23]. The gene encoding the key enzyme indole-3-pyruvate decarboxylase (IPDC), a decarboxylase that converts IPyA into indole-3-acetylaldehyde, has been identified and characterized in *Az. brasilense*, *Enterobacter cloacae*, and *Pseudomonas putida* [18, 25–27]. In these organisms, inactivation of this pathway resulted in lower IAA production, up to 90 % reduction in *Az. brasilense* [28], indicating the importance of the IPyA pathway in auxin production. However, the bacterial gene responsible for the aldehyde oxidation step of the IPyA pathway remains elusive. The presence of the IPyA pathway in some phytopathogens was proposed based on the identification of the corresponding intermediates [29, 30]. The IAM pathway is the best-characterized pathway in bacteria [31–33]. This two-step pathway is catalyzed by the enzymes tryptophan-2-monooxygenase (IaaM) and IAM hydrolase (IaaH), encoded by the *iaaM* and *iaaH* genes, respectively [23, 24]. The TAM pathway has been suggested in *B*. *cereus* and *Azospirillum* by the identification of tryptophan decarboxylase activity [34] and the conversion of exogenous tryptophan to IAA [35], but the genes involved in this pathway still need to be confirmed in bacteria. The biosynthesis of IAA via IAN has been extensively studied in plants, but it has not been characterized in bacteria. The steps leading to the conversation of tryptophan to IAN are still a matter of debate, but the last step of this pathway, converting IAN into IAA, was catalyzed by a nitrilase [36]. The possible IAN pathway in bacteria such as *Alcaligenes faecalis* was suggested due to the detection of nitrilase with specificity for indole-3-acetonitrile [37, 38]. Further, the nitrilase in *P. syringae* B728a is capable of hydrolyzing indole-3-acetonitrile to IAA, allowing this strain to use indole-3-acetonitrile as the nitrogen source [39]. Tryptophan-independent pathway was also suggested although no enzyme involved in this pathway has been characterized. In *Arabidopsis thaliana*, tryptophan-independent pathway was suggested based on the observation that tryptophan synthesis mutants increased IAA conjugates [40, 41]. In *Az. brasilense*, feeding experiments with labeled substrates revealed that 90 % of the IAA is synthesized via the tryptophan-independent pathway when tryptophan was absent in the medium [28].

Though much work has been performed on the IAA synthesis pathway in both plants and bacteria, as described above, the details of the biosynthetic pathways utilized by Gram-positive bacteria remain less clear. Stimulation of IAA production by tryptophan was previously described for Gram-positive bacteria [9]. In the phytopathogenic *Rhodococcus fascians*, the main biosynthetic route for IAA is the IPyA pathway, and the *ipdC* gene was functionally identified and expressed in *Paenibacillus polymyxa* E681 [42, 43]. In *B. amyloliquefaciens* FZB42, a fivefold increase in IAA secretion was recorded in the presence of tryptophan [9]. Deletion of the gene *ysnE*, which encodes a putative tryptophan acetyltransferase, results in a 72 % decrease of IAA production in FZB42 [9]. Meanwhile, an FZB42 mutant with a deletion of the putative nitrilase gene *yhcX* only produced 50 % of the wild type level of IAA [9]. These results indicated that the IAA synthesis in *B. amyloliquefaciens* FZB42 was tryptophan-dependent and that the IAN pathway and another uncharacterized pathway involving tryptophan acetyltransferase were the main routes for IAA biosynthesis [9].

The PGPR strain *B. amyloliquefaciens* SQR9 was isolated from cucumber rhizosphere (Table 1), and it showed efficient suppression of soil-borne *F. oxysporum* and protected the host from pathogen invasion [44–46]. Our previous study indicated that *B. amyloliquefaciens* SQR9 is an outstanding root colonizer with a population of 10^6^ CFU g^−1^ root in rhizoplane after 23 days of inoculation [44], which was a potential factor contributing to its ability to promote plant growth. A major antifungal antibiotic, bacillomycin D, and the global regulator AbrB were reported to be involved in the root colonization of *B. amyloliquefaciens* SQR9 [47, 48]. *B. amyloliquefaciens* SQR9 was shown to produce IAA, and this was suggested to contribute to its plant-growth-promoting effect [49]. Furthermore, addition of l-tryptophan in its growth medium significantly increased the yield of IAA [49]. Through genomic searching, the candidate genes potentially involved in the IAA biosynthesis pathways were obtained, and transcriptional responses to l-tryptophan and gene mutation analysis identified the key genes for IAA biosynthesis in SQR9. Moreover, a possible complete IPyA pathway was cloned and successfully expressed in both *B. amyloliquefaciens* SQR9 and *B. subtilis* 168.

**Table 1:** Strains and plasmids used in this study

Strains or plasmids	Description	Reference or source
*Escherichia coli*		
*E. coli* top10	F-mcrAΔ (mrr-hsdRMS-mcrBC) ψ80lacZΔM15Δ lacX74 nupG recA1 araD139Δ (ara-leu) 7697 galE15 galK 16 rpsL (StrR) end A1λ-	Invitrogen (Shanghai)
*B. amyloliquefaciens*		
SQR9	Wild-type isolate	[44]
ΔdhaS	*ΔdhaS*::Cm^r^	This study
ΔpatB	*ΔpatB*::Cm^r^	This study
ΔyclB	*ΔyclB*::Cm^r^	This study
ΔyclC	*ΔyclC*::Cm^r^	This study
ΔyhcX	*ΔyhcX*::Cm^r^	This study
ΔysnE	*ΔysnE*::Cm^r^	This study
patB-E	*B. amyloliquefaciens* SQR9 with pUBC19-P43-patB (kan^r^)	This study
yclC-E	*B. amyloliquefaciens* SQR9 with pUBC19-P43-yclC (kan^r^)	This study
dhaS-E	*B. amyloliquefaciens* SQR9 with pUBC19-P43-dhaS (kan^r^)	This study
SQR9-E	*B. amyloliquefaciens* SQR9 with pUBC19-P43-BCS (kan^r^)	This study
SQR9-CK	*B. amyloliquefaciens* SQR9 with pUBC19-P43 (kan^r^)	This study
*B. subtilis*		
168	Wild-type isolate	Lab strain
168-E	*B. subtilis* 168 with pUBC19-P43-BCS (kan^r^)	This study
168-CK	*B. subtilis* 168 with pUBC19-P43 (kan^r^)	This study
Plasmids		
pUBC19	Amp^r^ Km^r^; *B.subtilis*-*E. coli* shuttle vector	[47]
pNW33n	Cm^r^	[46]
pUBC19-P43	pUBC19 containing P43 promoter	This study
pUBC19-P43-patB	pUBC19 containing P43 promoter and *patB*	This study
pUBC19-P43-yclC	pUBC19 containing P43 promoter and *yclC*	This study
pUBC19-P43-dhaS	pUBC19 containing P43 promoter and *dhaS*	This study
pUBC19-P43-E	pUBC19 containing P43 promoter and fusion fragments for expression	This study
pUBC19-dhaS	pUBC19 containing *dhaS*	This study
pUBC19-yclC	pUBC19 containing *yclC*	This study
pUBC19-ysnE	pUBC19 containing *ysnE*	This study

*Cm* ^*r*^ chloroamphenicol resistant, *kan* ^*r*^ kanamycin resistant, *Amp* ^*r*^ Ampicillin resistant

## Results

### Screening of genes likely to be involved in IAA biosynthesis in the *B. amyloliquefaciens* SQR9 genome

Based on the proposed IAA biosynthesis pathways in plants and bacteria [23, 24], the entire *B. amyloliquefaciens* SQR9 genome was mined for genes involved in each step of IAA biosynthesis. The gene candidates were screened according to their deduced protein domains, which show putative enzyme activities already known in IAA metabolism (Table 2). In the IPyA pathway, genes encoding putative amino transferases including *patB*, *bioA* and *yotD*; the decarboxylase genes (*ydaP*, *pycA*, *padC*, *yclB*, and *yclC*) may be involved in the indolepyruvate decarboxylation reaction in the IPyA pathway and the tryptophan decarboxylation reaction in the TAM pathway. In both the IPyA and TAM pathways, the final step is the conversion of indole-3-acetaldehyde into IAA, which is catalyzed by indole-3-acetaldehyde dehydrogenase; in SQR9, the genes *aldX*, *dhaS*, *yfmT*, and *ywdH* may encode this enzyme. However, the genes coding for the putative amine oxidase in the first step of the TAM pathway were not detected in the SQR9 genome. For the possible IAN pathway, only the nitrilase gene (*yhcX*) was identified; the tryptophan acetyltransferase gene *ysnE*, which was involved in the uncharacterized tryptophan-dependent IAA biosynthesis pathway and was a main route in *B. amyloliquefaciens* FZB42 IAA biosynthesis [9], was also found in the SQR9 genome. The well-documented indolepyruvate decarboxylase *ipdC* gene in the IPyA pathway and the tryptophan monooxygenase (*iaaM*) and indole-3-acetamide hydrolase (*iaaH*) genes of the well characterized IAM pathway were not detected in the SQR9 genome [24].

**Table 2:** Possible *B. amyloliquefaciens* SQR9 genes involved in tryptophan dependent IAA production

IAA synthesis pathways	Enzymes proposed in each step	Putative genes in SQR9 and accession numbers^a^
IPyA	Amino transferase	*patB* (V529_31110), *bioA*(V529_17980), *yotD*(V529_21800)
	Indolepyruvate decarboxylase	*ydaP*(V529_04190), *pycA* (V529_14260), *padC*(V529_34370), *yclB*(V529_03390), *yclC*(V529_03400)
	IAAld dehydrogenase	*aldX*(V529_39560), *dhaS*(V529_19360), *yfmT*(V529_06950), *ywdH*(V529_29500)
IAM	Trp mono-oxygenase	Not detected
	IAM hydrolase	Not detected
TAM	Trp decarboxylase	*ydaP*(V529_04190), *pycA*(V529_14260), *padC*(V529_34370), *yclB*(V529_03390), *yclC*(V529_03400)
	Amine oxidase	Not detected
	IAAld dehydrogenase	*aldX*(V529_39560), *dhaS*(V529_19360), *yfmT*(V529_06950), *ywdH*(V529_29500)
IAN	Nitrilase	*yhcX*(V529_08860)
Other pathway	Tryptophan acetyltransferase	*ysnE*(V529_38080)

^a^Accession number was shown in bracket. *IPyA* ,* IAM*,* IAN* and* TAM* are abbreviates of indole-3-pyruvic acid, indole-3-acetamide, indole-3-acetonitrile, and tryptamine pathways, respectively

### Transcriptional responses to tryptophan identified the genes involved in tryptophan-dependent IAA biosynthesis of *B. amyloliquefaciens* SQR9

**Fig. 1:**
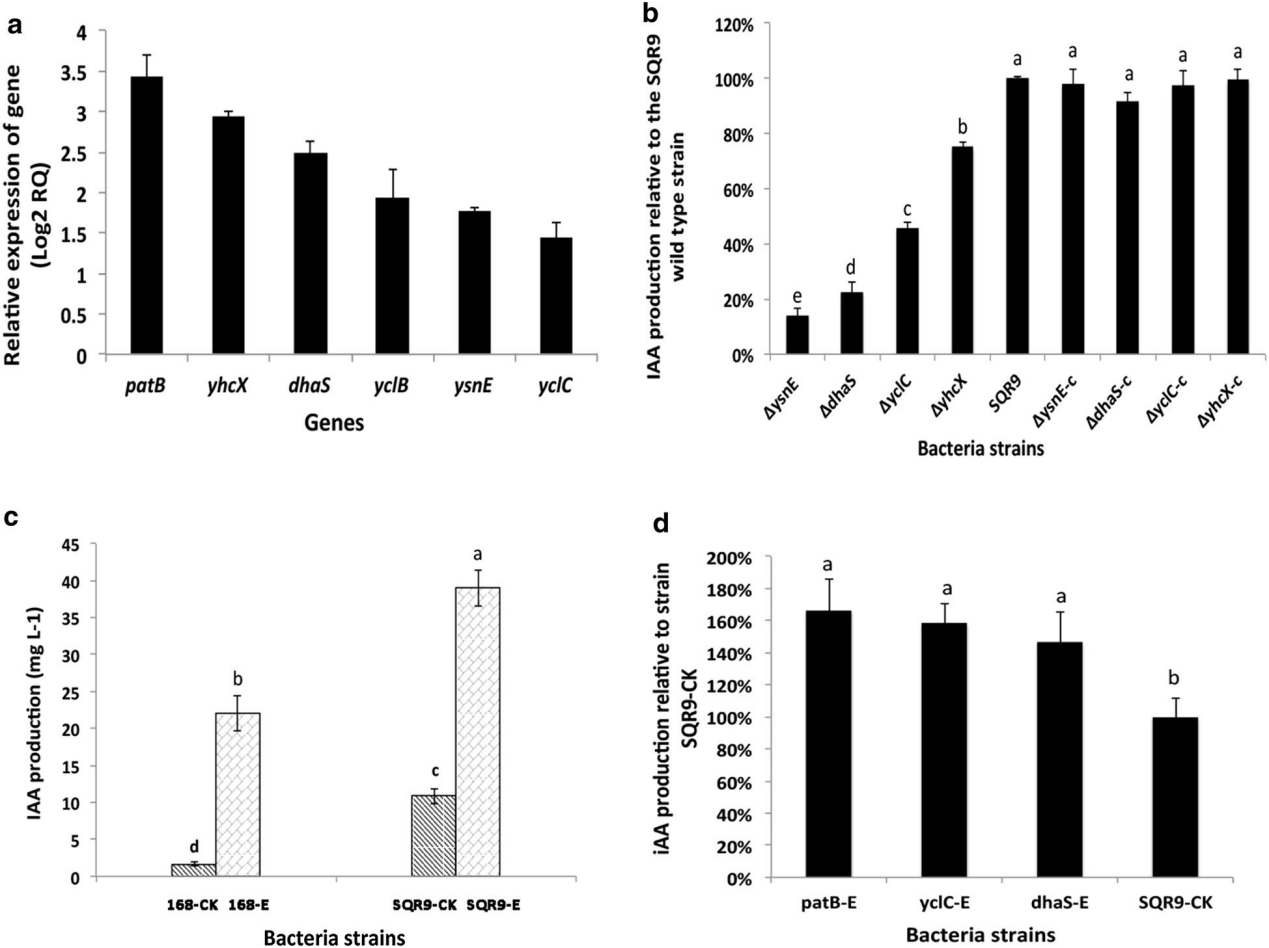
**a** Transcriptional levels of genes up regulated in the SQR9 grown with tryptophan relative to non-tryptophan treatment, as evaluated by qPCR. *B. amyloliquefaciens* SQR9 was grown in Landy medium with or without tryptophan for 65 h. The *recA* gene of SQR9 was used as an internal reference gene. *Bars* represent standard deviations of three biological replicates. **b** IAA production of the wild-type SQR9 and its derived strains. *Strains* were grown in Landy medium supplied with 3 mM tryptophan for 72 h. The bacterial strains ΔysnE-c, ΔdhaS-c, ΔyclC-c and ΔyhcX-c are complementary strains for the corresponding mutants. *Different letters* indicate significant differences (*P* < 0.05). **c** Quantification of the IAA produced by over-expression strains. Strains were cultured in Landy medium supplied with 3 mM tryptophan for IAA production. Bacteria strains are as follows: SQR9-E, pUBC19-P43-E in *B. amyloliquefaciens* SQR9; SQR9-CK, pUBC19-P43 in *B. amyloliquefaciens* SQR9; 168-E, pUBC19-P43-E in *B. subtilis* 168; and 168-CK, pUBC19-P43 in *B. subtilis* 168. *Different letters* indicate significant differences (*P* < 0.05). **d** Quantification of the IAA produced by sole gene expression strains. Strains were cultured in Landy medium supplied with 3 mM tryptophan for IAA production. Bacteria strains are as follows: patB-E, pUBC19-P43-patB in *B. amyloliquefaciens* SQR9; yclC-E, pUBC19-P43-yclC in *B. amyloliquefaciens* SQR9; dhaS-E, pUBC19-P43-dhaS in *B. amyloliquefaciens* SQR9; and SQR9-CK, pUBC19-P43 in *B. amyloliquefaciens* SQR9. *Different letters* indicate significant differences (*P* < 0.05)

Because all the gene candidates were potentially involved in the tryptophan-dependent IAA biosynthesis, the active genes were identified via the transcriptional responses of the screened genes upon the addition of tryptophan. When 3 mM tryptophan was added to the medium, SQR9 IAA production was increased 3.6-fold, indicating that IAA biosynthesis in SQR9 was tryptophan-dependent [49]. Six of the genes were found to be significantly induced by tryptophan (Fig. 1a): *patB*, encoding a conserved hypothetical protein predicted to be an aminotransferase, increased 3.5-fold; *yhcX*, a predicted nitrilase encoding gene, increased 3-fold; *dhaS*, encoding indole 3-acetaldehyde dehydrogenase, increased 2.5-fold; *ysnE*, a predicted tryptophan acetyltransferase encoding gene, increased twofold; *yclB*, encoding an aromatic-acid decarboxylase, increased 1.5-fold; and *yclC*, located in the same operon as *yclB*, encoding a UbiD family decarboxylase, increased twofold. These six genes were proposed to be involved in the tryptophan-dependent IPyA, TAM, and IAN pathways, as well as an uncharacterized IAA biosynthesis pathway, indicating that multiple IAA biosynthesis pathways existed in *B. amyloliquefaciens* SQR9. The other eight candidate genes, which were not induced by tryptophan (Additional file 1: Figure S1), were presumably not be involved in the tryptophan-dependent IAA biosynthesis in *B. amyloliquefaciens* SQR9.

### Gene knockout mutants revealed the key genes and pathways contributing to IAA biosynthesis of *B. amyloliquefaciens* SQR9

To confirm their contribution to IAA production in *B. amyloliquefaciens* SQR9, these six genes were individually deleted, and the IAA productions of these mutants were measured and compared with the wild-type SQR9 (Fig. 1b). All mutant strains showed growth curves comparable to that of wild-type SQR9. Indole-3-acetic acid was defined in SQR9 supernants by monitoring the transition of the parent molecular ion m/z 176 to the principle daughter ion m/z 130 in LC/MS analysis. Quantification of the IAA amounts present in culture filtrates of gene knockout mutants showed that strains ΔysnE, ΔdhaS, ΔyclC, and ΔyhcX produced only 14, 23, 45, and 76 % of the wild type SQR9 IAA production level, respectively (Fig. 1b), whereas strains ΔpatB and ΔyclB produced IAA at levels similar to wild-type SQR9. Complementation of the four mutants with the *B. amyloliquefaciens*- *Escherichia coli* shuttle vector pUBC19 carrying the corresponding deleted genes restored the IAA production (Fig. 1b). These results suggested that the pathway including tryptophan acetyltransferase was an important route for IAA biosynthesis in SQR9. The IPyA and TAM pathways also contributed substantially, but the IAN pathway only slightly contributed to IAA production in SQR9.

### Cloning and expression of the indole-3-pyruvic acid pathway and related genes for IAA biosynthesis

**Fig. 2:**
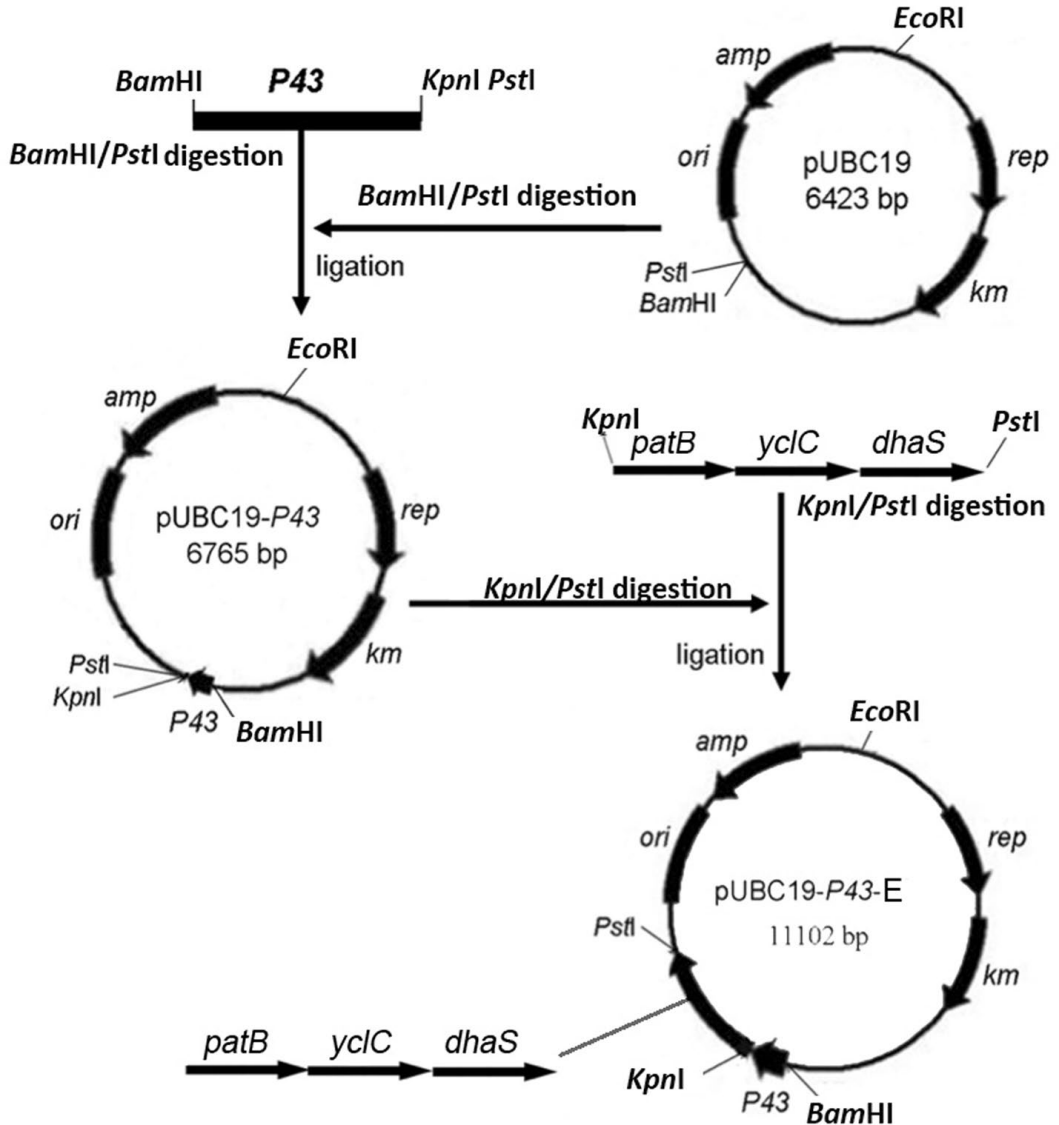
Construction of plasmids for expression. The *P43* promoter from *B. subtilis* 168 chromosomal DNA was introduced into the shuttle vector pUBC19 between the *Bam*HI and *Pst*I sites, and the recombinant plasmid was defined as pUBC19-P43. Genes for expression were PCR-amplified and were spliced by overlap extension. A *Kpn*I site was introduced into the forward primer of *patB*, and a *Pst*I site was introduced into the reverse primer of *dhaS*. The spliced fragment was ligated to the pUBC19-P43 plasmid in the *Kpn*I and *Pst*I sites, and the recombinant plasmid was named pUBC19-P43-E

From the above results, genes constituting a possible entire IPyA pathway were identified in SQR9: *patB*, predicted to encode an aminotransferase that catalyzes the conversion of tryptophan to indole-3-pyruvate acid, *yclC*, encoding an UbiD family decarboxylase involved in the conversion of indole-3-pyruvate acid to indole-3-acetaldehyde, and *dhaS*, encoding indole-3-acetaldehyde dehydrogenase to catalyze the last step of IPyA, converting indole-3-acetaldehyde to IAA. The three genes were separately or tandemly cloned into pUBC19 under the control of the *P43* promoter and then homologously expressed in strain SQR9 with the empty vector pUBC19-P43 served as control (Fig. 2). The potential IPyA pathway cluster was also heterologously expressed in strain *B. subtilis* 168 for further verification of its function since *B. subtilis* 168 showed much lower IAA production compared to that of SQR9 (Fig. 1c). Results showed IAA production of strains patB-E, yclC-E, dhaS-E were increased by 66.7, 58.8 and 47.1 % compared to SQR9-CK, respectively, when cultured in Landy medium supplemented with 3 mM tryptophan (Fig. 1d). Co-expression of the potential entire IPyA pathway cluster in SQR9 (strain SQR9-E) and *B. subtilis* 168 (strain 168-E) increased nearly 3.6 times (39 mg L^−1^) and 13.1 times (22 mg L^−1^) of IAA production compared with the empty vectors controls (SQR9-CK and 168-CK), respectively (Fig. 1c).

## Discussion

**Fig. 3:**
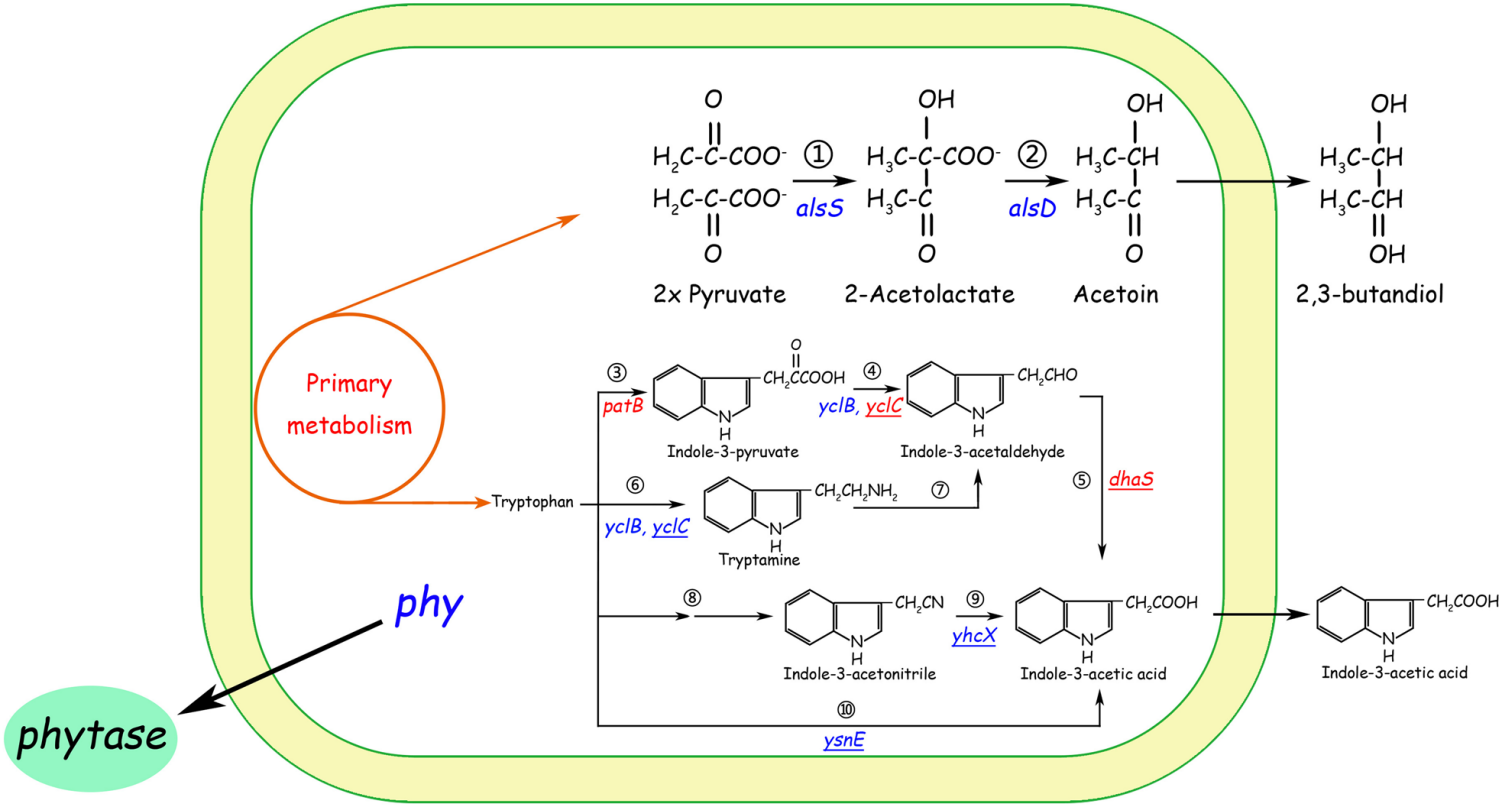
Factors contributed to plant-growth-promoting activities in SQR9. Indole-3-acetic acid (IAA), 2,3-butanediol and phytase synthesis are shown in the diagram. Genes are shown in italics. The acetolactate synthase AlsS ① and the decarboxylase AlsD ② catalyze the two-step conversion from pyruvate to acetoin. The following enzymes are proposed to be involved in tryptophan-dependent IAA biosynthesis in SQR9 based on a combination of chemical and genetic analyses: ③ tryptophan transaminase (PatB), ④ indole-3-pyruvate decarboxylase (YclC and YclB) and ⑤ indole-3-acetaldehyde dehydrogenase (DhaS) in the IPyA pathway; ⑥ tryptophan decarboxylase (YclC and YclB) and ⑦ amine oxidase in the TAM pathway; ⑧ oxidoreductase and acetaldoxime dehydratase and ⑨ nitrilase (YhcX) in the IAN pathway; and ⑩ tryptophan acetyltransferase (YsnE) in a predicted tryptophan-dependent IAA production pathway. The three genes shown in* red*, patB, yclC and dhaS, were predicted as a complete IPyA pathway for IAA synthesis, and both homologous expression in* B. amyloliquefaciens* SQR9 and heterologous expression in* B. subtilis* 168 successfully increased IAA production. The genes predicted to be involved in IAA synthesis in SQR9 were knocked out in this study. *Underlines* indicate that the generated knockout mutants produced less IAA than wild type

*B. amyloliquefaciens* SQR9 was isolated from cucumber rhizosphere with the capability to protect the host from pathogen invasion and promote the growth of cucumber [44]. Genomic analysis of *B. amyloliquefaciens* SQR9 revealed that several genes potentially contribute to its plant-growth-promoting effects. An entire set of *alsRSD* genes implicated in acetoin synthesis was identified. Moreover, the *phy* gene, which encodes phytase in *Bacillus* spp., was also identified in *B. amyloliquefaciens* SQR9. It was demonstrated that biosynthesis of phytase, acetoin and 2,3-butanediol in *B. amyloliquefaciens* SQR9 may affect its ability to promote plant growth (Fig. 3) [49]. In this study, we aimed to investigate the phytohormone IAA secreted by SQR9 and to identify possible IAA biosynthesis pathways in SQR9.

Taking advantage of the whole-genome sequence of SQR9, we used genomic scanning and related studies to investigate the key genes in the IAA biosynthesis pathways. The IPyA, TAM, and IAN pathways, as well as an uncharacterized pathway, were believed to participate in IAA biosynthesis of SQR9, according to the analysis of reverse transcription and of gene-knockout mutations. The uncharacterized IAA biosynthesis pathway appeared to be the most important route in SQR9 because IAA production by the *ysnE* gene knockout mutant was only 14 % of the wild type. The IPyA and TAM pathways also made substantial contributions, but the IAN pathway only contributed minorly IAA production in SQR9.

Genes involved in the IAM pathway, which has been described mainly in phytopathogenic bacteria, were not detected in the SQR9 genome. This two-step pathway has been reported for other PGPR strains. The IAN pathway has been suggested in *Al. faecalis* [37, 38] and *P. syringae* B728a [39] and was believed to contribute to IAA production in *B. amyloliquefaciens* FZB42 [9]. In *B. amyloliquefaciens* FZB42, YhcX was predicted to act as a nitrilase based on its similarity to nitrilase 2 of *A. thaliana*, which catalyzes the final step of the IAN pathway [50]. The *yhcX* gene was also detected in the SQR9 genome, but knockout of this gene only led to a 24 % decrease in IAA production. IAA production in the *ysnE* gene mutant was only 14 % of the wild type, but this specific metabolic process is poorly studied. The predicted product of the *ysnE* gene belongs to the N-acetyltransferase superfamily of various enzymes that catalyze the transfer of an acyl group to a nitrogen atom on the acceptor molecule. In *B. amyloliquefaciens* FZB42, this protein is similar to a putative tryptophan acetyltransferase gene localized within the tryptophan biosynthesis gene cluster in *Az. brasilense* and has been predicted to participate in tryptophan-dependent IAA production [9, 51, 52], while in *Bacillus* strains, this gene locates away from the tryptophan biosynthesis gene cluster. We deduced that the *ysnE* gene product is one of the most important intermediates in *B. amyloliquefaciens* strains, and in-depth study of the role of this gene in IAA biosynthesis will have great significance.

Most beneficial bacteria produce IAA via the IPyA pathway [24]. In our study, gene products of *patB*, *yclC* and *dhaS* were proposed to constitute the entire IPyA pathway in SQR9. Both homologous expression in *B. amyloliquefaciens* SQR9 and heterologous expression in *B. subtilis* 168 successfully led to increased IAA production with tryptophan supplied in the medium. In *Bacillus* strains, the *patB* gene was defined as a putative aminotransferase in the aspartate aminotransferase family. This family belongs to the pyridoxal phosphate (PLP)-dependent aspartate aminotransferase (AAT) superfamily, whose members catalyze four types of reactions: transamination (movement of amino groups), racemization (redistribution of enantiomers), decarboxylation (removing COOH groups), and various side-chain reactions. The major groups in this conserved domain correspond to aspartate aminotransferase a, b, and c and to other amino acids, including aromatic amino acids, such as tryptophan. A previous study constructed two AAT mutants of *Rhizobium meliloti* and revealed that neither was essential for the biosynthesis of IAA in the absence of exogenous tryptophan but both contributed to IAA biosynthesis when high levels of exogenous tryptophan were present [53]. The *yclC* gene, located in the same operon as *yclB*, was considered a hypothetical protein, possibly encoding a decarboxylase from the UbiD family, which contains several different decarboxylases. The gene product of *yclC* was predicted to be an indole-3-pyruvate decarboxylase, which has no significant similarity with the reported indole-3-pyruvate decarboxylase in other organisms. Neither do the *yclC* gene has significant similarity with recent reported flavin monooxygenase YUCCA1 that directly converts IPyA to IAA in *Arabidopsis* [54–56]. The gene responsible for the aldehyde oxidation step of the IPyA pathway in bacteria remains elusive. DhaS, similar to aldehyde dehydrogenase, which likely catalyze the final reaction of the IPyA pathway, has not been considered to be involved in IAA synthesis in *B. amyloliquefaciens* FZB42 [9, 51]. However, in SQR9, the gene product of *dhaS* is demonstrated to be involved in IAA synthesis because IAA production of this gene knockout mutant was only 23 % of the wild type. The differences between SQR9 and FZB42 strains, which belong to the same species, might be caused by individual differences, as strain SQR9 could produce much more IAA with tryptophan supplied than strain FZB42 reported by Idris et al. [9]; IAA produced by mutant strains of FZB42 were quantified without tryptophan supplied, although very few, bacteria synthesis tryptophan from indole itself [57], while in our study, IAA production was always quantified with tryptophan supplied [9].

The *patB* gene was up regulated in reverse-transcription analysis, but its gene deficient mutant strain did not show lower IAA production, and the over expression experiment confirmed that this gene participates in the IPyA pathway. This suggests that although it responds to tryptophan sensitively, the gene product of *patB* might not be the solely gene responsible for the aminotransferase reaction. The *yhcX* gene encoding the putative nitrilase was markedly up regulated in the reverse-transcription analysis, whereas ΔyhcX only showed a 24 % decrease in IAA production. These confusing phenomena might be explained as follows: (1) it is possible that strain SQR9 possesses several different isoforms of the enzyme for IAA synthesis, each able to utilize multiple substrates, as Kittell et al. [53] reported that aromatic amino acid aminotransferase can act upon all three aromatic amino acids. Furthermore, although *ipdC*-encoded indolepyruvate decarboxylases from some bacteria have high affinity for indolepyruvate, they can also utilize phenylpyruvate, pyruvate and benzoylformate as substrates [42, 58–60]. (2) As reported for *B. megaterium*, several genes that encode isozymes of glucose dehydrogenase are known [61], and the aminotransferase and nitrilase steps contain multiple enzymes acted as isozymes. When the *patB* and *yhcX* genes were inactivated, other genes functioned as isozymes. In bacteria, nitrilases belong to a large family. Branch I Nitrilases are known to have true nitrilase activity [62, 63], which may be implicated in IAA biosynthesis. (3) The gene product of *patB* acts as an aminotransferase, converting tryptophan to indole-3-pyruvic acid, suggesting that *patB* gene product is not only a likely immediate precursor for IAA biosynthesis but also a precursor for indolelactic acid (ILA) and tryptophol (Tol), which have been identified as products of physiological origin in *Agrobacterium tumefaciens* [30]. (4) There are different pathways to synthesize IAA in bacteria, the break down of one pathway could enhance the others. Thus, we concluded that the primary roles of the genes identified in this study might not be the production of IAA; rather, their involvement in the IAA synthesis pathway is incidental. Furthermore, we speculate that each step of the IAA synthesis pathway might contain several isozymes. Therefore, the roles of the reactions catalyzed by the six gene products in IAA biosynthesis in SQR9 remain to be elucidated. Feeding experiments, double and triple mutant analysis, appropriate labeling studies and identification of biosynthetic intermediates will be required to uncover the metabolic route for tryptophan-dependent IAA synthesis in SQR9.

## Conclusions

In conclusion, the results presented here identified six genes involved in IAA biosynthesis of *B. amyloliquefaciens* SQR9 by transcription and IAA quantification analysis of the relevant mutants. The predicted tryptophan-dependent IAA biosynthesis pathways in SQR9 were shown in Fig. 3, and pathways such as IAN, TAM, IPyA and an uncharacterized pathway, which was supposed to be the most important one, are suggested to appear in this strain. Moreover, a possible IPyA pathway for IAA biosynthesis, consisting of *patB*, *yclC* and *dhaS*, was confirmed through homologous and heterologous expression in *B. amyloliquefaciens* SQR9 and *B. subtilis* 168. This study deepened our understanding of IAA biosynthesis in *B. amyloliquefaciens* SQR9 and provided molecular evidence for genetic modification of *Bacillus* spp. for better agricultural application. The overexpression strain of SQR9 produced more IAA and this strain has the potential to be more efficiently to promote plant growth.

## Methods

### Strains and growth conditions

The strains and plasmids used in this study are described in Table 1. *B. amyloliquefaciens* SQR9 (China General Microbiology Culture Collection Center, CGMCC accession no. 5808) was isolated from the rhizosphere of cucumber. *E. coli* top10 was used as the host for all plasmids, and *B. subtilis* 168 was used as the host for heterologous expression of the IAA biosynthesis pathway genes. *B. amyloliquefaciens* SQR9 and *B. subtilis* 168 were grown at 30 °C in low-salt Luria–Bertani (LLB) medium (peptone, 10 g L^−1^; yeast extract, 5 g L^−1^; NaCl, 5 g L^−1^) solidified with 1.5 g 100 mL^−1^ agar; when necessary, 5 μg mL^−1^ chloramphenicol (Cm) or 20 μg mL^−1^ kanamycin (Km) was added. *E. coli* top10 was grown in LLB medium at 37 °C; when required, Cm was added to a final concentration of 12.5 μg mL^−1^ or Km to 30 μg mL^−1^. For IAA production, the bacteria were grown for 72 h in Landy medium [64] containing 3 mM l-tryptophan at 25 °C and 90 rpm.

### Genome analysis of genes involved in IAA production in SQR9

Based on the whole-genome nucleotide sequence of SQR9 (NCBI accession no. CP006890), the function of all protein-coding genes in SQR9 against reference genomes of *B. amyloliquefaciens* FZB42 [51] and *B. subtilis* 168 [65] was obtained by using BLASTP (parameters: *e*-value: 1e-5, coverage >60 %, identity >50 %). The genes were identified according to their deduced protein domains, which show putative enzyme activities already known in IAA metabolism.

### Reverse transcription analysis of genes involved in IAA synthesis

RNA was isolated from *B. amyloliquefaciens* SQR9 cells fermented in Landy medium with or without 3 mM l-tryptophan at 65 h post-inoculation. The cells were harvested by centrifugation at 4 °C (10 min, 5000×*g*). Total RNA samples were extracted using an RNAiso Plus kit (TaKaRa, Dalian, China) according to the manufacturer’s protocol. RNA was reverse-transcribed into cDNA in a 20-μL reverse transcription system (TaKaRa, Dalian, China) according to the manufacturer’s instructions.

Transcription levels of genes potentially involved in SQR9 IAA biosynthesis with or without tryptophan in the growth medium were measured by quantitative reverse transcription PCR (qRT-PCR) using a SYBR Premix Ex Taq kit (TaKaRa, Dalian, China). The primers used to amplify these genes are listed in Transcription analysis part of Additional file 2: Table S1. The *recA* gene of SQR9 was served as an internal control. Reactions were carried out on an ABI 7500 system under the following conditions: cDNA was denatured for 10 s at 95 °C, followed by 40 cycles of 5 s at 95 °C and 34 s at 60 °C. The 2^−ΔΔCT^ method was used to analyze the qRT-PCR data.

### Gene knockout by allelic exchange

To delete the genes of *B. amyloliquefaciens* SQR9, fragments of approximately 1 kb in length both upstream and downstream of each target gene were amplified. The 1.1-kb chloramphenicol resistance gene (Cm^r^) was amplified from plasmid pNW33n with primers, which were partially overlapped with the upstream and downstream fragments of each target gene. Upstream and downstream fragments were fused with the chloramphenicol resistance gene by overlap extension. Primers used in this experiment are shown in Gene knockout part of Additional file 2: Table S1. The 25 µl mixture for the first step of the overlapping PCR is as follows: 13.2 µL water, 5 µL PrimeSTAR buffer (5×), 2 µL dNTP mix (2.5 mM each), 1.5 µL (15 ng) upstream fragment, 1.5 µL (15 ng) downstream fragment, 1.5 µL (15 ng) Cm^r^ fragment, and 0.3 µL PrimeSTAR HS DNA polymerase. The PCR program was as follows: 98 °C for 2 min, 98 °C for 10 s, 56 °C for 10 s and 72 °C for 3.5 min, 15 cycles. The second step of overlapping PCR, the mixture included 16.7 µL water, 5 µL PrimeSTAR buffer, 2 µL dNTP mix, 1 µL forward primer for the upstream fragment, 1 µL reverse primer for the downstream fragment, 1 µL unpurified product from the first PCR step, and 0.3 µL PrimeSTAR HS DNA polymerase. The PCR program was as follows: 98 °C for 2 min, 98 °C for 10 s, 52 °C for 10 s and 72 °C for 3.5 min, 32 cycles. All PCR products were gel-purified using an AxyPrep DNA gel purification and extraction kit (Axygen, Hangchow, USA).

The fused fragments for the target genes were individually transformed into competent cells of *B. amyloliquefaciens* SQR9 [41], and the transformants were selected on LLB agar plates containing 5 μg mL^−1^ Cm. The correct mutants were verified by PCR using the primer sets in Mutant verification part of Additional file 2: Table S1.

### Complementation of the disrupted genes

Plasmid pUBC19-X (X represents genes of *dhaS*, *yclC*, *yhcX* and *ysnE*) were constructed to complement each deleted gene in SQR9 mutants. Primers used in this study are shown in Complementary experiment part of Additional file 2: Table S1. The *dhaS* and *ysnE* genes (including their promoters), bordered by primer-introduced *Pst*I and *Bam*HI sites, were amplified from SQR9 genomic DNA using the primers dhaScF/dhaScR and ysnEcF/ysnEcR. The *yclC* and *yhcX* genes (except for their promoters) bordered by primer-introduced *Pst*I and *Kpn*I sites (yclCcF/yclCcR and yhcXcF/yhcXcR) were amplified. After purification and digestion with the corresponding restriction enzymes, the fragments of *dhaS* and *ysnE* were cloned into the *B. amyloliquefaciens*-*E.coli* shuttle vector pUBC19; *yclC* and *yhcX* gene fragments were ligated into the pUBC19-P43 vector, which is described in a subsequent section, under the control of the *P43* promoter from *B. subtilis* 168 for their promoters remain uncertain. Plasmids were sequenced to confirm their fidelity and then transformed into each corresponding mutant strain of SQR9 to obtain complemented strains.

### IAA production analysis

Wild-type and gene knock-out mutants of SQR9 and *B. subtilis* 168 strains were propagated overnight in 3 mL Landy medium, and then, 1 ml aliquots were transferred into 100 mL Landy medium supplied with 3 mM l-tryptophan. After incubation for 72 h, the density of each culture was measured spectrophotometrically at 600 nm, each of the cell culture was adjusted to OD_600_ = 5.0 with sterilized water and then the cells of SQR9, *B. subtilis* 168 and mutant strains were removed by centrifugation (5000×*g*, 20 min, 4 °C). Next, 50 µL culture supernatant of each strain was subjected to ELISA analysis with the IAA ELISA KIT (Cloud-Clone Crop. USA), and the IAA content was quantified. Liquid cultures (100 mL) of each strain were adjusted to pH 2.5 with 1.0 M HCl and extracted three times with ethyl acetate (1:3, v/v). The organic solvents were vacuum-dried at 37 °C and then dissolved in 3 ml methanol. The extract samples were filtered through a 0.45-µm membrane before analysis. Extracted samples were separated with Reverse Phase High Performance Liquid Chromatography (RP-HPLC) using a reversed-phase C18 analytical column (9.4 by 150 mm; Agilent Technologies, Santa Clara, CA). The mobile phase was methanol-0.1 % acetic acid (60/40) at a flow rate of 0.4 mL min^−1^ for 25 min at 220 nm using a UV detector. IAA was quantified by integrating the areas under peaks with the help of authentic IAA from Sigma. The molecular weight of IAA identified by RP-HPLC was determined by an Agilent 6410 triple quadrupole LC/MS apparatus (Agilent Technologies). The electrospray needle was operated at a spray voltage of 4.5 kV and a capillary temperature of 300 °C. The mobile-phase components were methanol-0.1 % acetic acid (60/40) at a flow rate of 0.4 ml min^−1^. Data acquisition was done in the positive ion mode.

### Expression of the identified genes or indole-3-pyruvic acid pathway cluster of SQR9

Primers P43F/P43R (Additional file 2: Table S1) were used to clone the *P43* promoter sequence from *B. subtilis* 168 chromosomal DNA; the *Bam*HI site was introduced into the forward primer P43F, *Kpn*I and *Pst*I sites were introduced into the reverse primer P43R. The P43 fragment was inserted into the shuttle vector pUBC19 between the *Bam*HI and *Pst*I sites, and the recombinant plasmid was designated as pUBC19-P43 (Fig. 2). Genes for expression were PCR-amplified with the primers shown in Additional file 2: Table S1. For single gene expression, a *Kpn*I site was introduced into the forward primer and a *Pst*I site was introduced into the reverse primer of each gene. For gene cluster expression, a *Kpn*I site was introduced into the forward primer of *patB*, and a *Pst*I site was introduced into the reverse primer of *dhaS*. The PCR fragments for gene cluster expression were spliced by overlap extension described above. Gene fragments and the pUBC19-P43 plasmid were digested with *Kpn*I and *Pst*I and then ligated together, and the recombinant plasmids were named pUBC19-P43-patB, pUBC19-P43-yclC, pUBC19-P43-dhaS and pUBC19-P43-E (Fig. 2).

Plasmids pUBC19-P43-patB, pUBC19-P43-yclC and pUBC19-P43-dhaS were transformed into the competent cells of *B. amyloliquefaciens* SQR9 [41] to obtain the strains patB-E (pUBC19-P43-patB in SQR9), yclC-E (pUBC19-P43-yclC in SQR9) and dhaS-E (pUBC19-P43-dhaS in SQR9). Plasmids pUBC19-P43-E and pUBC19-P43 (as empty vector control) were transformed into the competent cells of SQR9 and *B. subtilis* 168 [66] to obtain the strains SQR9-E (pUBC19-P43-E in SQR9), SQR9-CK (pUBC19-P43 in SQR9), 168-E (pUBC19-P43-E in *B. subtilis* 168), and 168-CK (pUBC19-P43 in *B. subtilis* 168). Transformants were grown in LLB medium containing 20 µg mL^−1^ km. For the detection of IAA production, transformants were incubated in Landy medium supplied with tryptophan, and the cultures were analyzed with HPLC and ELISA as described above.

### Nucleotide sequence accession numbers

The GenBank accession numbers for the genes in IAA biosynthesis in the SQR9 genome (NCBI accession no. CP006890) are shown in Table 2. The GenBank accession numbers for the *phy* gene and the *alsRSD* gene cluster are V529_21880, V529_35850, V529_35860 and V529_35870, respectively.

## Additional files

Additional file 1:
**Figure S1.** Relative transcription of genes. Transcriptional levels of genes in the SQR9 grown with tryptophan relative to non-tryptophan treatment evaluated by qPCR. *B. amyloliquefaciens* SQR9 was grown in Landy medium with or without tryptophan for 65 h. The *recA* gene of SQR9 was used as an internal reference gene. Bars represent the standard deviations of three biological replicates. Additional file 2:
**Table S1.** Primers used in this study. Primers used in this study were listed in this table.
