# Supplementary figures and images for: Analysis and cloning of the synthetic pathway of the phytohormone indole-3-acetic acid in the plant-beneficial Bacillus amyloliquefaciens SQR9
Source: Microb Cell Fact. 2015 Sep 4;14:130. doi: 10.1186/s12934-015-0323-4 (PMC4558970; doi:10.1186/s12934-015-0323-4)

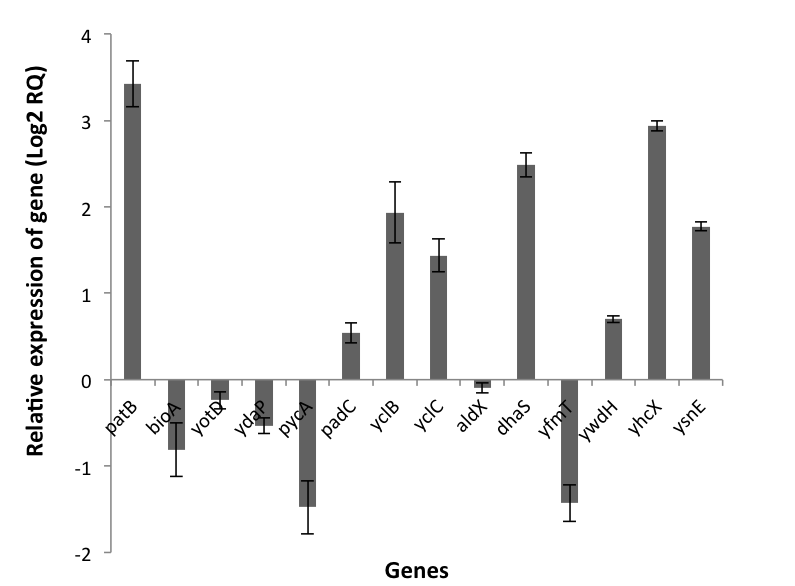

Supplement: Additional file 1: — Figure S1. Relative transcription of genes. Transcriptional levels of genes in the SQR9 grown with tryptophan relative to non-tryptophan treatment evaluated by qPCR. B. amyloliquefaciens SQR9 was grown in Landy medium with or without tryptophan for 65 h. The recA gene of SQR9 was used as an internal reference gene. Bars represent the standard deviations of three biological replicates. [file 12934_2015_323_MOESM1_ESM.png]
