# Supplementary material for: Analysis and cloning of the synthetic pathway of the phytohormone indole-3-acetic acid in the plant-beneficial Bacillus amyloliquefaciens SQR9
Source: Microb Cell Fact. 2015 Sep 4;14:130. doi: 10.1186/s12934-015-0323-4 (PMC4558970; doi:10.1186/s12934-015-0323-4)
Supplement: Additional file 2: — Table S1. Primers used in this study. Primers used in this study were listed in this table. [file 12934_2015_323_MOESM2_ESM.doc]

**Table S1 Primers used in this study**

| Experiment | Primer name | | Sequence 5’-3’^a^ |  |
| --- | --- | --- | --- | --- |
| Transcription analysis |  | |  |  |
|  | dhaSF | | GCTCGTCAGTCAAGAACAGCA |  |
|  | dhaSR | | TCATCCACATCCGCAAATACA |  |
|  | patBF | | ATGCGCTTCCGATGTGG |  |
|  | patBR | | CGGCTTCTCTTGTGTTTTCGT |  |
|  | yclBF | | GGCAACGGGAGCGATATTC |  |
|  | yclBR | | GGAGACACGACAAGATGGGTTT |  |
|  | yclCF | | TTCCACGAAAACGCGCTAC |  |
|  | yclCR | | CGACATCTTCATCCACGACAA |  |
|  | yhcXF | | AACATCATCGGCGGCTCT |  |
|  | yhcXR | | CCACCATTTCCGCTCATTC |  |
|  | ysnEF | | AGTAACTAAACTAGAGTGGCA |  |
|  | ysnER | | CGACGCAGTCGGACTGCAA |  |
|  | aldXF | | ATGAACAATCTCGACACCTGGA |  |
|  | aldXR | | GGAGCCATCGTCAGCATAAAC |  |
|  | padCF | | ATGCCCGCGCTGTGAATCG |  |
|  | padCR | | AGCAGGTTTGGCATTGGTA |  |
|  | pycAF | | CACTGAAGATCCGCTGAATGA |  |
|  | pycAR | | ACGAGCAGTGAATCATAGTA |  |
|  | yfmTF | | ACAGACTGTACAGAATAC |  |
|  | yfmTR | | CTTCATGAGGCTTCAGCA |  |
|  | yhxAF | | ACGGCAGACGGTATTTGGA |  |
|  | yhxAR | | GCGGGTTGATGGCTTTG |  |
|  | ywdHF | | GGCAGCTATCACGGATTTGAC |  |
|  | ywdHR | | AAGAAGGATAACGGAAAGCGAAG |  |
|  | recAF | | AAAAAACAAAGTCGCTCCTCCG |  |
|  | recAR | | CGATATCCAGTTCAGTTCCAAG |  |
| Gene knockout |  | |  |  |
|  | dhaS upF | | ACCATTACGAACAGATTACA | |
|  | dhaS upR | | ATCGTTAAAGAACTCATTCGTA | |
|  | dhaS downF | | AGTGTCTGGATCAATCTGGA | |
|  | dhaS downR | | AGAGAGGTCTCTGTTCTCA | |
|  | patB upF | | ATGACAAGGTTTATCAGACACA | |
|  | patB upR | | CGTCAGGAGACGTATAAC | |
|  | patB downF | | CATGATGAAGCCTGACGC | |
|  | patB downR | | AGTGCAGCCGATGAGAAGA | |
|  | yclB upF | | AGAGGCGATGTCATTCAG | |
|  | yclB upR | | ATGACGAGCTTCATATTGA | |
|  | yclB downF | | AGGAGGAATCTGATAATG | |
|  | yclB downR | | ACTCGGATTGATCGTATA | |
|  | yclC upF | | AATATGAAGCTCGTCATCGGA | |
|  | yclC upR | | ACCTCTTCGTCGACTGTTA | |
|  | yclC downF | | AGGATGAGGTTGCAATGA | |
|  | yclC downR | | ATCGGTCTTATTTCGATCA | |
|  | yhcX upF | | AGATACAGAAACCCATGAATA | |
|  | yhcX upR | | CTTCCATATTCCGTATGACCA | |
|  | yhcX downF | | ATCGAAATGGTCGTGATCG | |
|  | yhcX downR | | ATTGCCGTGATAGCCTCTGTA | |
|  | ysnEupF | | ATCCTGCAATACGATCATC | |
|  | ysnEupR | | ATGACGTTAAACATAGACTGA | |
|  | ysnE downF | | CATGCTCTTCAGATGATCC | |
|  | ysnE downR | ACGGTGACCATATGGATGGA | | |
|  | Cm-dhaSF | CGGGAGGTTTGTTACGAATGAGTTCTTTAACGATGCATAAAGTGTAAAGCCTGGGG | |  |
|  | Cm-dhaSR | TCCAGATTGATCCAGACACTTTTCACTTCTGTATAATGTGGAATTGGGAACGGAAA | |  |
|  | Cm-patBF | GACCACGGCATATTCGGTTATACGTCTCCTGACGGCATAAAGTGTAAAGCCTGGGG | |  |
|  | Cm-patBF | CCAGATCAAATAGGAAGCGTCAGGCTTCATCATGAATGTGGAATTGGGAACGGAAA | |  |
|  | Cm-yclBF | AGCGTAAAGGAGGGGTCAATATGAAGCTCGTCATGCATAAAGTGTAAAGCCTGGGG | |  |
|  | Cm-yclBR | TAAAATCCTGATAGGCCATTATCAGATTCCTCCTAATGTGGAATTGGGAACGGAAA | |  |
|  | Cm-yclCF | AAGAAGGGCAGCTCTTAACAGTCGACGAAGAGGTGCATAAAGTGTAAAGCCTGGGG | |  |
|  | Cm-yclCR | CAGCGCGGGCATGTTTTCATTGCAACCTCATCCTAATGTGGAATTGGGAACGGAAA | |  |
|  | Cm-yhcXF | TTTGAAAAGAAAATGGTCATACGGAATATGGAAGGCATAAAGTGTAAAGCCTGGGG | |  |
|  | Cm-yhcXR | CGATCACGACCATTTCGATATTCGGATTCGTTTAATGTGGAATTGGGAACGGAAA | |  |
|  | Cm-ysnEF | CAGAAAAGGGGTTTCAGTCTATGTTTAACGTCATGCATAAAGTGTAAAGCCTGGGG | |  |
|  | Cm-ysnER | CATTGCGCTTTTGCAGGATCATCTGAAGAGCATGAATGTGGAATTGGGAACGGAAA | |  |
| Mutant verification |  |  | |  |
|  | ysnEvF | | ACTGGAGTCAGACATCCA |  |
|  | ysnEvR | | GTCTATCTCATCGGATTG |  |
|  | yhcXvF | | ACACGTGATGTCTTCTGCA |  |
|  | yhcXvR | | ATGACTTCATCACTGATCA |  |
|  | dhaSvF | | AAAGTTTTTCCCCTTGTCATTAATA |  |
|  | dhaSvR | | AACATTCTCTGGTATTTGGACTC |  |
|  | yclCvF | | TCCTTCAGCTCTTGTACCTCGATAA |  |
|  | yclCvR | | GCGACGGAGAGTTAGGTTATTGG |  |
|  | patBvF | | ATCGTCCTGCTGTGCAGCT |  |
|  | patBvR | | GCAAGCTCGCTGACGACG |  |
|  | yclBvF | | ACTCACAGACAACCTGAAG |  |
|  | yclBvR | | ATCTCTCCTTCCAGAACG |  |
| Complementary experiment |  | |  |  |
|  | dhaScF | | AACTGCAGAATCGATAAGCTTATTGTA |  |
|  | dhaScR | | CGGGATCCCACACTCGCTCAACTCCTTC |  |
|  | yclCcF | | AACTGCAGATCTGATAATGGCCTATCA |  |
|  | yclCcR | | CGGGATCCCAACCTCATCCTTTCTTA |  |
|  | yhcXcF | | AACTGCAGGTCTGAGAAACTTGATCTGA |  |
|  | yhcXcR | | CGGGATCCTTATTTCAGGTGCGTAATCCG |  |
|  | ysnEcF | | AACTGCAGCTATCCCGCCTTACATTTTCA |  |
|  | ysnEcR | | CGGGATCCGTTACAATTATTATACCA |  |
| Gene expression |  | |  |  |
|  | patBseF | | GGGGTACCACATACATGAACTTTGATC |  |
|  | patBseR | | AACTGCAGTTTCCGCTGCTAAGCGAGCGC |  |
|  | yclCseF | | GGGGTACCGGAGGAATCTGATAATGG |  |
|  | yclCseR | | AACTGCAGTTATTGATTCATTAAGTC |  |
|  | dhaSseF | | GGGGTACCACGAATGAGTTCTTTAACGA |  |
|  | dhaSseR | | AACTGCAGCTGACAGGCAGCAGTTTC |  |
| Expression of gene cluster |  | |  |  |
|  | yclCeF | | AGAATGAAAGCAGCGCTCGCTTAGCAGCGGAAAGGGAGGAATCTGATAATGG | |
|  | yclCeR | | CTTTTTGTGATCTGCATCGTTAAAGAACTCATTCGTCTTATTGATTCATTAAGTC | |
|  | patBeF | | GGGGTACCACATACATGAACTTTGATC | |
|  | patBeR | | CTTTCCGCTGCTAAGCGAGCGC | |
|  | dhaSeF | | ACGAATGAGTTCTTTAACGA | |
|  | dhaSeR | | AACTGCAGCTGACAGGCAGCAGTTTC | |
|  | P43F | | CGCGGATCCTGATAGGTGGTATGTTTTCGC | |
|  | P43R | | AACTGCAGGGTACCCTTGGTCAAGTTGCGCATGTGTACATTCCTCTCTT | |

^a^All DNA sequences are written in a 5' to 3' orientation. Restriction enzyme sites are underline
